# *Commiphora leptophloeos* Bark Decoction: Phytochemical Composition, Antioxidant Capacity, and Non-Genotoxic Safety Profile

**DOI:** 10.3390/ph18060863

**Published:** 2025-06-10

**Authors:** José Rafael da Silva Araujo, Rafael de Felício, Camila Marinho da Silva, Palloma Lima de Oliveira, Silvany de Sousa Araújo, Laís Roberta Deroldo Sommaggio, Adriana Fabiana Corrêa da Silva, Paulo Henrique Valença Nunes, Bruno Oliveira de Veras, Erwelly Barros de Oliveira, Jaciana dos Santos Aguiar, Maria Aparecida Marin-Morales, Daniela Barretto Barbosa Trivella, Ana Maria Benko-Iseppon, Márcia Vanusa da Silva, Ana Christina Brasileiro-Vidal

**Affiliations:** 1 Departamento de Genética, Universidade Federal de Pernambuco, Recife 50670-901, Brazil; rafael.silvaa@ufpe.br (J.R.d.S.A.);; 2 Laboratório Nacional de Biociências, Centro Nacional de Pesquisa em Energia e Materiais, Campinas 13083-100, Brazil; rafael.felicio@lnbio.cnpem.br (R.d.F.);; 3 Departamento de Biologia, Universidade Estadual Paulista, Rio Claro 13506-752, Brazil; 4 Departamento de Bioquímica, Universidade Federal de Pernambuco, Recife 50670-901, Brazil; 5 Departamento de Antibióticos, Universidade Federal de Pernambuco, Recife 50670-901, Brazil

**Keywords:** Salmonella test, imburana-de-cambão, MTT, micronuclei test, molecular network, UPLC-MS/MS phytochemical

## Abstract

**Background:** *Commiphora leptophloeos* has long been used in Latin American folk medicine for the treatment of respiratory and gastrointestinal disorders. Therefore, toxicological and phytochemical investigations are required to assess the safety and support the evidence-based use of its bark in medicinal applications. This study aimed to evaluate the aqueous bark extract of *C. leptophloeos*, focusing on its chemical composition and its antioxidant, cytotoxic, and genotoxic properties. **Methods**: The aqueous extract was obtained by decoction of dried bark samples. Phytochemical characterization was conducted using ultra-performance liquid chromatography coupled with tandem mass spectrometry (UPLC-MS/MS), and data were processed using the NP^3^ MS Workflow 1.1.4 software, allowing for the annotation of key secondary metabolites. Antioxidant activity was assessed through multiple in vitro assays, including DPPH, ABTS, phosphomolybdenum, and reducing power tests. Cytotoxicity was evaluated using the MTT assay, while genotoxicity was investigated through the Ames test and micronucleus assay. **Results**: Phytochemical analysis revealed several flavonoids, with procyanidin B2 annotated as a major compound. The extract exhibited strong antioxidant activity, with EC_50_ values of 5.43 μg/mL (DPPH), 12.40 μg/mL (ABTS), 35.20 μg/mL (phosphomolybdenum), and 31.27 μg/mL (reducing power). The MTT assay showed no cytotoxic effects at concentrations up to 6400 μg/mL. Furthermore, both the Ames and micronucleus assays showed the absence of genotoxic effects at concentrations up to 1600 μg/plate and 400 μg/mL, respectively. **Conclusions**: The aqueous bark extract of *C. leptophloeos* demonstrates strong antioxidant potential and a favorable safety profile, with no detectable cytotoxicity or genotoxicity at concentrations effective in antioxidant assays. Further studies are recommended to confirm and validate its traditional medicinal properties using appropriate in vivo models, followed by pre-clinical evaluations.

## 1. Introduction

The *Commiphora leptophloeos* (Mart.) J.B.Gillet (syn. *Bursera martiana* Engl.; *Bursera orinocensis* Engl.; *Bursera leptophloeos* Mart.), a member of the Burseraceae family, is a medicinal plant commonly known as imburana, imburana-de-cambão, and umburana [1]. This species is endemic to South America and native to Bolivia, Brazil, and Venezuela [2,3]. The bark of *C. leptophloeos* is traditionally used by various local communities in the form of decoctions (tea) or syrup to treat respiratory diseases, such as flu, cough, and bronchitis. It is also employed for its anti-inflammatory properties [4,5,6,7]. In addition to the bark, other parts of the plant, including leaves, fruits, flowers, and latex, are used in traditional medicine to treat similar conditions, as well as to promote wound healing and relieve stomach pain and gastritis [6,8,9].

Despite its widespread traditional use, the chemical composition of the bark of *C. leptophloeos* remains relatively underexplored. Several compounds have been identified in bark extracts prepared using organic solvents such as ethanol, methanol, and chloroform. These include phenolic acids, such as gallic acid, chlorogenic acid, and protocatechuic acid, as well as flavonoids like catechin and quinic acid. Additionally, the presence of the bioactive lignan hinokinin has also been reported [10,11]. On the other hand, aqueous extractions have revealed the presence of condensed tannins, including proanthocyanidins such as prodelphinidin, profisetinidin, and procyanidin, along with phenolic acids like gallic acid, and flavonoids such as catechin and epicatechin [12].

More comprehensive characterizations, particularly those using UPLC-MS/MS systems and dereplication analysis, are essential for advancing the phytochemical understanding of *C. leptophloeos* bark extracts. These methods are crucial for identifying key bioactive compounds and elucidating the pharmacological potential of this promising botanical resource.

In addition, the safety of medicinal plants must be assessed through toxicological analysis, following guidelines established by international regulatory agencies [13,14,15,16]. Among these, in vitro genotoxicity tests are particularly relevant, including the cytokinesis-block micronucleus assay and the bacterial reverse mutation assay (Ames test). These assays are recommended by regulatory authorities such as the U.S. Food and Drug Administration (FDA) [17] and the European Medicines Agency (EMA) [18]. The cytokinesis-blocking micronucleus assay may be used to evaluate the ability of plant-derived compounds to induce chromosomal damage during at least one cell division. Such damage is detected through cytogenetic biomarkers, including micronuclei, nuclear buds, and nucleoplasmic bridges [19]. On the other hand, the reverse mutation test using *Salmonella typhimurium* strains deficient in histidine synthesis assesses the mutagenic potential of a sample by determining its ability to cause point mutations, such as base pair substitutions, deletions, or insertions, that restore the bacteria’s ability to grow in histidine-deficient media [20].

To support the potential therapeutic applications of *C. leptophloeos* in phytotherapy, the present study aimed to address the following questions: (1) Does the extract of *C. leptophloeos* neutralize free radicals and reduce metal ions, and if so, at what concentrations? (2) Which compounds and groups of secondary metabolites are present in the aqueous extract *of C. leptophloeos*? (3) Do the bark extracts of *C. leptophloeos* induce in vitro cytotoxicity and genotoxicity in normal human cells and mutagenicity in Salmonella bacterial strains?

## 2. Results

### 2.1. Phytochemical Evaluation

**Figure 1:**
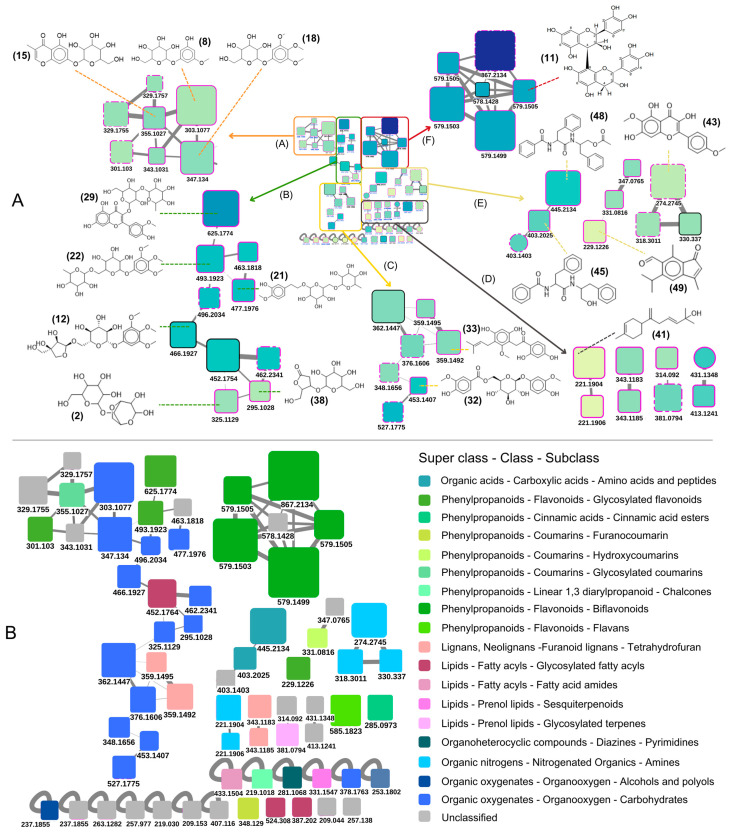
(**A**) Spectra Similarity Molecular Network (SSMN) of the [M+H]^+^ compounds detected in spectra of the aqueous extract of *Commiphora leptophloeos* by UPLC-MS/MS. The network was constructed using output files from the NP^3^ MS Workflow and visualized with Cytoscape v. 3.9.1. Each node (circles or squares) represents a consensus spectrum, which in this network is pointed out as the [M+H]^+^ ion (or a putative metabolite). Nodes are connected based on the similarity of their mass fragmentation spectra, forming clusters that might represent compounds sharing chemical similarity (e.g., from the same chemical family). Colors represent a range of *m*/*z* values (yellow = 200 Da, dark blue = 867 Da). Geometric forms and borders indicate the database source and quality of the chemical annotation. For a detailed description of the legend used, refer to Supplementary Figure S1. The letters A to F represent the clusters. Each *m*/*z* value is shown below the respective node in the SSMN, and annotated nodes are described in more detail in Table 1. (**B**) Class, superclass, and pathway of metabolites annotated in the [M+M]^+^ SSMN of the aqueous extract of *Commiphora leptophloeos* by UPLC-MS/MS. The different colors represent the classification of the compounds. Annotations from the GNPS database were considered in this analysis.

The phytochemical analysis (performed in triplicate) of the aqueous extract of *C. leptophloeos* bark detected 410 ions, which were distinctly separated by the NP^3^ MS Workflow 1.1.4 software (Supplementary Figure S1). Of these, 67 were identified as protonated compounds ([M+H]^+^—putative compounds), all of which were retained after filtering for those consistently present in all three replicate analyses. Subsequently, 51 compounds were investigated in more detail after an additional filtering step based on occurrence (peak area). The corresponding chromatograms obtained from the UPLC-MS/MS analyses can be observed in Supplementary Figure S2. These compounds, represented as nodes in the Spectra Similarity Molecular Network (SSMN), were grouped into six components (clusters of structurally related compounds based on mass spectra fragmentation similarity) and thirteen non-components (self-loop nodes) (Figure 1 and Supplementary Figure S1). A total of 22 compounds were annotated using GNPS or UNPD-ISDB databases. Among the annotated metabolites, the flavonoid procyanidin B2 (*m*/*z* 579.1450—Supplementary Figure S3) was the most recurrent compound (Figure 1A and Table 1).

Regarding the clusters (Figure 1A), a large cluster primarily composed of organooxygen compounds was annotated. This cluster was further subdivided into three subclusters (A, B, and C), as follows.

Subcluster A consisted of monoglycosylated compounds from the organooxygen class. Annotated compounds included: (3-hydroxy-5-methoxy-phenyl)-beta-D-glucopyranoside (**8**; *m*/*z* 303.1077), 5,7-dihydroxy-3-methylchromone-7-O-beta-D-glucoside (**15**; *m*/*z* 355.1027), and 1-beta-D-glucopyranosyloxy-3,4,5-trimethoxybenzene (**18**; *m*/*z* 347.1340).

Subcluster B comprised diglycosylated compounds of the same organooxygen class, such as (3,4-dimethoxyphenoxy)-6-[(3,4,5-trihydroxyoxan-2-yl)oxymethyl]oxane-3,4,5-triol (**12**; *m*/*z* 466.1927); forsythoside (**21**; *m*/*z* 477.1976), 1-(alpha-L-rhamnopyranosyl-(1->6)-beta-D-glucopyranosyloxy)-3,4,5-trimethoxybenzene (**22**; *m*/*z* 493.1923), and 3-O-beta-D-glucopyranosyl-2-deoxy-D-ribono-gamma-lactone (**38**; *m*/*z* 295.1028). This subcluster also included diglycosylated flavonoids, such as isorhamnetin 3-O-neohesperidoside (**29**; 625.1774).

Subcluster C included phenolic and chalcone compounds, such as 6′-O-vanilloyltachioside acid (**32**; *m*/*z* 453.1407) and the chalcone glycophenone (**33**; 34; *m*/*z* 359.1402 and *m*/*z* 359.1495).

A group of very small clusters, each composed of one or two [M+H]^+^ nodes, was referred to as cluster D. In this group, only the sesquiterpenoid helianthol A was annotated (**41**; *m*/*z* 221.1904).

Additionally, group E comprised three small clusters, each containing three nodes, and included the flavonoid butoletol (**43**; *m*/*z* 331.0816), the dipeptide aurantiamide (**45**; *m/z* 403.2025), the carboxylic acid benzenepropanamide N-[2-(acetyloxy)-1-(phenylmethyl)ethyl]-alpha-(benzoylamino) (**48**; *m*/*z* 445.2134), and the sesquiterpenoid 3,7-dimethyl-5-isopropyl-6-formylindenone (**49**; *m*/*z* 229.1226).

Finally, cluster F was a pure spectra similarity cluster composed of six nodes, all annotated as bioflavonoids. Among them, procyanidin B2 was annotated with high confidence (Supplementary Table S2). This compound was annotated at different retention times (**14**; 19; 28), forming three nodes in the SSMN. Based on the peak area, procyanidin B2 was the most abundant compound in the aqueous extract of *C. leptophloeos* bark, representing 4.76% of all spectra detected. In self-loop nodes, two additional compounds were annotated: megastigman (3S,5R,8R)-3,5-dihydroxy-6,7-megastigmadien-9-one 5-O-beta-D-glucopyranoside (**23**; *m*/*z* 387.202) and the cinnamic acid (+)-(E,E)-3-hydroxy-7-phenyl-4,6-heptadienic acid (**30**; *m*/*z* 219.1018).

Although not all ions were annotated by the databases, it was possible to annotate a wide variety of metabolite classes. Using GNPS, a total of 18 subclasses were assigned to 11 broader metabolite classes in the aqueous extract of *C. leptophloeos*, with organooxygen compounds being the most abundant, followed by flavonoids and lignans (Figure 1B). In contrast, analysis via the UNPD database enabled the identification of 30 distinct metabolite classes, with flavonoids emerging as the most frequently detected, followed by organooxygen compounds (Supplementary Table S1).

It is important to mention that the annotation data details for the substances highlighted in the text above are presented in Supplementary Table S2.

### 2.2. Antioxidant Potential

**Figure 2:**
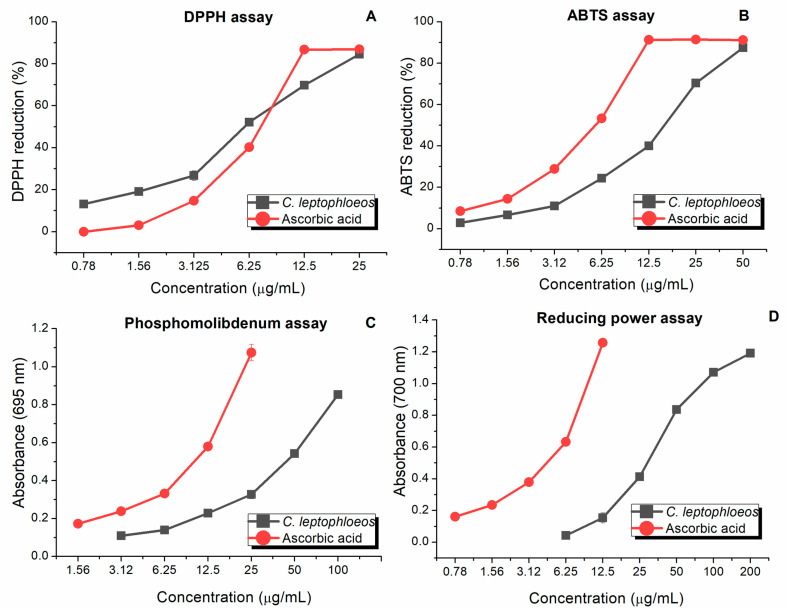
Average antioxidant activity of the aqueous extract of *Commiphora leptophloeos* bark. DPPH (**A**) and ABTS (**B**) results expressed as percentage scavenging, and phosphomolybdenum assay (**C**) and reducing power (**D**) results expressed as absorbance values. Ascorbic acid was used as a reference standard. All experiments were carried out in triplicates (*n* = 3). Error bars represent the standard deviation.

All tested concentrations of the aqueous extract of *C. leptophloeos* in the DPPH, ABTS, phosphomolybdenum, and reducing power assays demonstrated concentration-dependent effects (Figure 2). In the DPPH assay, the extract neutralized free radicals at rates ranging from 13.16 to 84.51%, while the ascorbic acid standard ranged from 3.00 to 86.87% (Figure 2A). In the ABTS assay, neutralization by the extract ranged from 2.84 to 87.46%, compared to 8.55% to 91.11% for ascorbic acid (Figure 2B). Regarding the phosphomolybdenum assay, absorbance values for the extract ranged from 0.11 and 0.83 nm, while that of ascorbic acid ranged from 0.17 to 1.07 nm (Figure 2C). In the reducing power assay, absorbance values for the extract ranged from 0.04 to 1.191 nm, and those of the ascorbic acid ranged from 0.161 to 1.256 nm.

The EC_5z0_ values of the aqueous extract, determined from the dose–response curves, were: 5.43 μg/mL (DPPH), 12.40 μg/mL (ABTS), 31.27 μg/mL (reducing power), and 35.20 μg/mL (phosphomolybdenum tests) (Table 2). The corresponding EC_50_ values for ascorbic acid were 6.40, 5.48, 12.70, and 6.39 μg/mL, respectively. Statistical comparison (*t*-test) revealed no significant difference between the extract and ascorbic acid only for DPPH assay (Table 2).

### 2.3. Cytotoxicity

**Figure 3:**
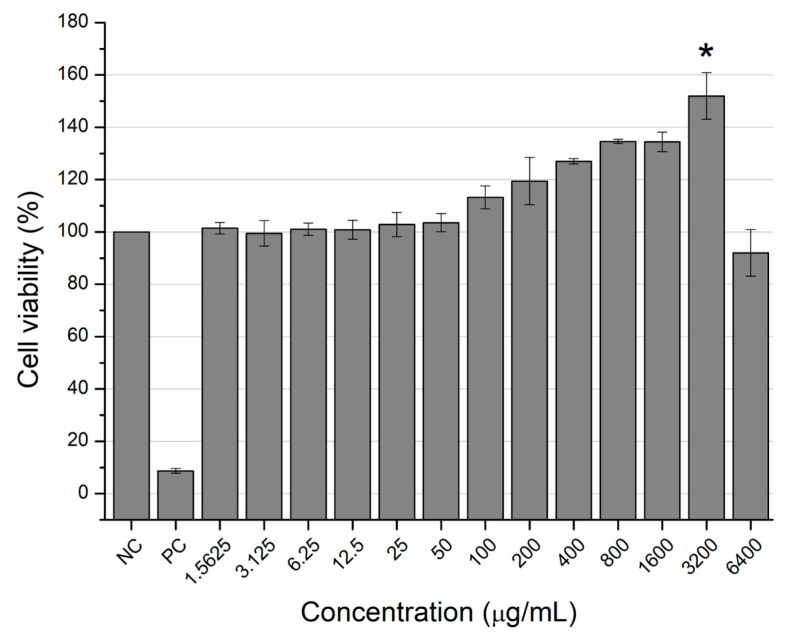
Cytotoxicity of the aqueous extract of *Commiphora leptophloeos* bark assessed by the MTT test using murine fibroblast L929 cell line. Values represent the mean and standard deviation of the percentage of cell viability (*n* = 4). NC: Negative Control; PC: Positive Control. * Statistical difference in comparison to the NC by the Tukey parametric test (*p* < 0.05).

Cytotoxicity analysis after 24 h of exposure to the aqueous extract of *C. leptophloeos* bark revealed that none of the tested concentrations induced cytotoxicity in murine fibroblast cells (L929 cell line), as no significant reduction in cell viability was observed in the MTT assay. However, the 3200 μg/mL concentration statistically increased the cell viability, reaching 177.30% compared to the control (Figure 3).

### 2.4. Genotoxicity

Regarding the total alterations, none of the concentrations (6.25 to 400 μg/mL) of the aqueous extract of *C. leptophloeos* bark induced a significant increase in the number of alterations (ranging from 16.55 to 21.55 alterations/1000 cells) compared to the negative control (18.00 alterations/1000 cells), as assessed by the CBMN test. This suggests the absence of genotoxic effects in terms of overall chromosomal damage. However, when individual types of alterations were analyzed, a significant increase in micronucleus frequency was observed at the highest concentration (400 μg/mL), with 15.65 micronuclei/1000 cells. None of the tested concentrations significantly affected the cell cycle progression, which is consistent with the absence of cytotoxic effects observed in the MTT assay (Table 3).

### 2.5. Ames/Salmonella Test (Salmonella/Microsome Assay)

The Ames test was performed using *Salmonella typhimurium* strains TA100 and TA98, both in the absence and presence of metabolic activation (−S9 and +S9, respectively). The aqueous extract of *C. leptophloeos* bark, tested at concentrations ranging from 6.25 to 1600 μg/plate, did not induce a statistically significant increase in the number of revertant colonies compared to the negative control for both strains (Table 4). Additionally, the Mutagenicity Index for all extract treatments remained below 2, further supporting the absence of mutagenic potential.

## 3. Discussion

In the present study, the aqueous bark extract of *C. leptophloeos* demonstrated antioxidant potential, with no evidence of cytotoxicity or genotoxicity at the tested concentrations, based on multiple recommended assays. Furthermore, several secondary metabolites from the extract were annotated via UPLC-MS/MS, contributing to a better understanding of its biological relevance.

Previous studies have reported the presence of metabolites restricted to the flavonoids class in the aqueous bark extract of *C. leptophloeos*, particularly proanthocyanidins such as prorobinetinidin, prodelfinidin, and profisetinidin [21]. In the present study, procyanidin B2 (**11**; **19**; **28** *m*/*z* 579.1450) was annotated as the predominant metabolite in *C. leptophloeos* bark. This compound belongs to the B-type proanthocyanidins, which are composed of flavan-3-ol units such as (+)-catechin and (−)-epicatechin linked by a single bond, typically between the carbon 4 (C4) of the upper “extension” unit and carbon 8 (C8) or 6 (C6) of the lower or “starter” unit [22]. The presence of polymeric procyanidins in *C. leptophloeos* bark was first reported by Trentin et al. [21] and later confirmed by Dantas-Medeiros et al. [11,23], who identified B-type dimers in hydroethanolic bark extracts. Procyanidin B2, along with the other compounds annotated in *C. leptophloeos* bark in this study, is discussed in the context of the antioxidant, cytotoxic, and genotoxic profiles analyzed in the conducted bioassays.

The antioxidant activity of the aqueous extract of *C. leptophloeos* bark was comparable to that of the ascorbic acid standard in the DPPH assay, with EC_50_ of 5.43 and 6.40 μg/mL, respectively, indicating a relevant antioxidant potential. Although the extract exhibited antioxidant capacity similar to that of pure ascorbic acid in the DPPH assay, such comparisons should be interpreted with caution. Differences in compound purity, solubility, and synergistic interactions inherent to complex mixtures may influence the dose–response outcomes and potentially lead to an overestimation of the extract’s potency. Nevertheless, distinct plant parts can exhibit different biological effects. For instance, Cordeiro et al. [24] reported antioxidant activity of the aqueous leaf extract of *C. leptophloeos*, with 58–70% DPPH neutralization at concentrations of 50–250 μg/mL. On the other hand, the aqueous bark extract in the present study achieved 52–84% neutralization at considerably lower concentrations (6.25–25 μg/mL), suggesting that the bark extract has greater antioxidant potential than the leaf extract.

Procyanidin B2, the major compound annotated in the bark of *C. leptophloeos*, is likely one of the main contributors to its observed antioxidant activity. When evaluated as a purified compound, it exhibits a potent antioxidant effect, with an EC_50_ of 0.6942 μg/mL in the DPPH assay, which helps explain the low EC_50_ value (5.43 μg/mL) observed for the aqueous bark extract [25]. Procyanidin B2 is also present in green tea (*Camellia sinensis* (L.) Kuntze), a medicinal plant widely recognized for its strong antioxidant properties [26,27]. Another compound annotated in the extract, isorhamnetin 3-O-neohesperidoside (29; *m*/*z* 625.1774), classified as a flavonoid (UNPD), also previously demonstrated antioxidant potential. When isolated from the aqueous leaf extract of *Acacia salicina* Lindl., this metabolite showed significant activity in the lipid peroxidation assay at a concentration of 189 μg/mL [28], suggesting that it may also contribute to the antioxidant effects observed in *C. leptophloeos* bark.

Although flavonoids are the major contributors to the antioxidant activity observed in the *C. leptophloeos* bark extract, low-abundance compounds may also have a significant role. Coumarins, for instance, exhibit notable free radical scavenging activity due to their catechol groups and a 2H-chromen-2-one core, which facilitates electron delocalization [29]. Hydroxycinnamic acids, such as ferulic and caffeic acid derivatives, act as chain-breaking antioxidants by donating electrons or hydrogen atoms, with their effectiveness influenced by hydroxyl substitution patterns on the aromatic ring [30]. Lignans contribute both through direct antioxidant effects and by modulating endogenous defenses through activation of the Nrf2 transcription factor [31]. Additionally, sesquiterpenoids, though present in low amounts, have demonstrated strong ROS scavenging potential [32]. Therefore, even in small quantities, these secondary metabolites may exert additive or synergistic effects, enhancing the extract’s overall antioxidant capacity and reflecting the phytochemical complexity of the species.

Flavonoids, isocoumarins, and their glycosylated derivatives, identified among the classes of secondary metabolites in the aqueous extract of *C. leptophloeos* bark, have been extensively reported in the literature for their anti-inflammatory, antioxidant, and antidiabetic properties [33,34,35]. These bioactivities provide pharmacological support for the traditional use of the species in treating inflammatory conditions and metabolic disorders, reinforcing the ethnopharmacological relevance of the extract. However, further experimental tests are essential to directly evaluate the anti-inflammatory activity of the *C. leptophloeos* extract and to provide concrete evidence to support its traditional use.

It is important to note that these chemical-based tests primarily evaluate free radical scavenging or reducing capacity in simplified, non-biological systems. While useful for preliminary screening, such assays do not fully replicate the complexity of oxidative stress modulation in biological environments. Therefore, additional mechanistic studies—such as assessing intracellular ROS levels or the modulation of endogenous antioxidant enzymes (e.g., SOD, CAT, GPx)—are recommended to better understand the antioxidant potential of the extract in biological systems. This limitation should be considered when interpreting the antioxidant findings of the present study.

Regarding cytotoxicity, the present study demonstrated that the aqueous bark extract exhibited no cytotoxic effects on L929 mouse fibroblast cells at concentrations up to 6400 μg/mL. The concentrations used in MTT assay were selected based on the EMA guidelines [18], which recommend testing concentrations close to or above 5000 μg/mL to ensure the evaluation of compounds present in low abundance. Similarly, previous studies have shown that the ethanolic leaf extract of *C. leptophloeos* also exhibited no cytotoxicity in Raw 264.7 macrophage cells [11].

On the other hand, concentrations between 100 and 3200 μg/mL increased viability, with the 3200 μg/mL concentration showing a statistically significant effect. A similar trend was reported for the ethanolic leaf extract of *C. leptophloeos*, where concentrations of 250 and 500 μg/mL increased viability of NIH/3T3 murine fibroblast cells by approximately 30% [24]. Additionally, the hydroalcoholic leaf extract of *C. leptophloeos* significantly increased the viability of Raw 264.7 murine macrophage cells by 60% at a concentration of 100 μg/mL, without altering the cell cycle, as shown by flow cytometry [11]. In the present study, the increase in cell viability observed in the MTT assay at intermediate concentrations was not accompanied by changes in the cell cycle, as indicated by the CBPI results from the micronucleus assay (6.25 to 400 μg/mL), further supporting the absence of cytotoxic effects across all tested concentrations.

Glycosylated compounds, commonly found in aqueous extracts, may contribute to increased cell viability by serving as energy sources [36]. In the present study, 13 compounds classified as carbohydrates were annotated via GNPS, including (3-hydroxy-5-methoxy-phenyl)-beta-D-glucopyranoside (**8**; *m*/*z* 303.1077), 1-β-D-glucopyranosyloxy-3,4,5-trimethoxybenzene (**18**; *m*/*z* 347.134), 3-O-β-D-glucopyranosyl-2-deoxy-D-ribono-gamma-lactone (**38**; *m*/*z* 295.1028), 6′-O-vanilloyltachioside (**32**; *m*/*z* 453,1407), and forsythoside E (**21**; *m*/*z* 477.1976). Additionally, flavonoids have also been associated with enhanced cell viability, as previously observed in leaf extracts of *Elaeis guineensis* Jacq [37]. In the present work, the flavonoids procyanidin B2 (**14**, **19**, and **28**) and 1-(alpha-L-rhamnopyranosyl-(1->6)-beta-D-glucopyranosyloxy)-3,4,5-trimethoxybenzene (**22**; *m*/*z* 493.1923) were annotated and may have contributed to the increased cell viability observed. The increased viability may be related to the antioxidant activity of procyanidin B2, which has demonstrated the ability to mitigate oxidative stress and enhance cell viability in various cell models [38,39,40,41,42].

Among the metabolites annotated from LC-MS/MS analysis of *C. leptophloeos* bark, some stand out for their previous lack of cytotoxicity in various cell types and concentration ranges. For instance, magnolioside (**15**; *m*/*z* 355.1027) showed no cytotoxic effects at concentrations below 100 μM in SK-OV-3 ovarian cancer cells [41]. Similarly, 1-beta-D-glucopyranosyloxy-3,4,5-trimethoxybenzene (**18**; *m*/*z* 347.134) was non-cytotoxic at 10 μg/mL in JB6 mouse epidermal cells [42]. Isorhamnetin 3-O-neohesperidoside (**29**; *m*/*z* 625.1774) also exhibited low cytotoxicity, with an IC_50_ of 75.6 μg/mL in bone-marrow-derived macrophages from C57B/L mice [40]. These findings support the evidence that the annotated metabolites are non-toxic at the tested concentrations, reinforcing the absence of cytotoxicity observed for the aqueous bark extract of *C. leptophloeos* in the present study.

The absence of cytotoxicity observed in L929 fibroblasts exposed to aqueous bark extract of *C. leptophloeos* may reflect the favorable safety profile of both major and minor constituents. Although many of these compound classes exhibit cytotoxic effects in cancer models, they generally show low toxicity in non-tumorigenic cells. Coumarins, for example, selectively modulate cellular pathways without affecting the viability of healthy cells [43]. Similarly, cinnamic acid derivatives maintain cell viability above 50% in BEAS-2B and MCF-10A cells at concentrations up to 50 μM [44], and sesquiterpenoids like atractylon show low toxicity in primary human mononuclear cells and Vero cells [45]. These findings suggest that the lack of cytotoxicity of the extract may not be attributed solely to flavonoid content but also to the synergistic or mild effects from other constituents.

Regarding genotoxicity, the in vitro micronucleus test (CBMN) and the bacterial reverse mutation assay (Ames test) are sensitive and widely accepted methodologies, required by regulatory agencies such as the Food and Drug Administration (FDA) [17], European Medicines Agency (EMA) [18], and Brazilian Health Regulatory Agency (ANVISA) [46] to assess the safety of new products.

In the CBMN assay, the aqueous bark extract of *C. leptophloeos* did not induce a statistically significant increase in nuclear alterations, such as micronuclei, nuclear buds, or nucleoplasmic bridges, in L929 murine fibroblasts. Consistent with these results, previous in vivo studies showed that oral administration of 2000 mg/kg of *C. leptophloeos* extract did not lead to increased micronuclei formation or DNA fragmentation, as evaluated by the CBMN and comet assays, respectively, in mouse erythrocytes [12].

Previous studies on the major compound, procyanidin B2, have shown it to be non-genotoxic in both in vivo and in vitro models. Takahashi et al. [47] reported no micronuclei formation in mouse bone marrow cells at doses up to 2000 mg/kg. Similarly, chromosomal aberration tests using Chinese hamster lung (CHL) cells revealed no structural changes, indicating an absence clastogenic activity and supporting the safety of procyanidin B2.

Additionally, isorhamnetin 3-O-neohesperidoside (I3ON), a glycosylated flavonoid from Acacia salicina, has shown potent antioxidant and antigenotoxic activity in cellular studies. Bouhlel et al. [28] reported that I3ON inhibits lipid peroxidation and protects against hydroxyl-radical-induced DNA damage, while modulating antioxidant (HMOX2 and TXNL) and DNA repair (e.g., XPC, POLD1, PCNA, LIG4) genes, indicating a robust cellular response to oxidative stress. Although direct evidence of Nrf2 activation by I3ON is limited, studies on its aglycone, isorhamnetin, show it promotes Nrf2 nuclear translocation in HepG2 cells, enhancing antioxidant gene expression (e.g., HO-1, GCL) and glutathione levels, providing protection against t-BHP-induced oxidative stress [48]. Furthermore, isorhamnetin has also mitigates benzo[a]pyrene (B[a]P)-induced genotoxicity in HepG2 cells by promoting RAD51 expression via miR-34a suppression, aiding in DNA double-strand break repair. These findings suggest that I3ON contributes to the antioxidant and genoprotective effects observed in *C. leptophloeos* extracts, supporting its safety and therapeutic potential.

Although some sesquiterpenoids, such as helenalin and hymenoxon, have documented genotoxic effects, this study found that the aqueous bark extract of *C. leptophloeos* did not induce genotoxicity in L929 fibroblasts. Genotoxicity reported in the literature is often linked to mechanisms like DNA alkylation and ROS generation, but these effects depend on specific chemical structures, concentrations, and exposure times [49]. In the evaluated extract, sesquiterpenoids are present at low levels, which may explain the absence of harmful effects observed in our assays. This supports the hypothesis that compounds with known genotoxic potential, when present in complex natural matrices at subcytotoxic doses, may not compromise cellular integrity. These findings reinforce the extract’s safety and therapeutic potential.

In the bacterial reverse mutation assay using *S. typhimurium*, strain TA98 carries a deletion in the histidine operon at the hisD3052 allele. This strain is specifically designed to detect reversions caused by mutagenic agents that induce frameshift mutations [50]. In the present study, treatment with the aqueous extract of *C. leptophloeos* did not result in a statistically significant increase in the number of revertant colonies compared to the negative control in TA98, indicating the absence of frameshift mutation. Similarly, strain TA100, which contains a mutation in the hisG46 allele and can detect both base-pair substitutions and frameshift mutations [50], also showed no increase in revertants. These results indicate that the extract does not induce substitution mutations either. Supporting these findings, isolated procyanidin B2, annotated in this study, has also been reported as non-genotoxic in the Ames tests using both TA98 and TA100 strains of *S. typhimurium* at concentrations of 56, 313, 625, 1250, 2500, and 5000 μg/plate [47], reinforcing the absence of genotoxic effects associated with the extract.

No published Ames test results are available for the other listed compounds. However, flavonoids and phenolic glycosides are generally not mutagenic in bacterial assays. Isorhamnetin, a methylated flavonol, has shown significant antimutagenic activity in the *Salmonella typhimurium* TA98 strain exposed to nitroarenes such as 2-nitrofluorene (2-NF), 3-nitrofluoranthene (3-NFA), and 1-nitropyrene (1-NP). At 1 μmol/plate, isorhamnetin reduced mutagenicity by 43.6%, 60%, and 45.6%, respectively, without causing bacterial toxicity, suggesting a specific protective effect rather than general cytotoxicity. These nitroarenes are environmental mutagens found in diesel exhaust and charred foods, and their inhibition by isorhamnetin underscores the potential of dietary flavonoids in reducing genotoxic risk [51]. The efficacy of the compound may stem from its hydroxyl and methoxyl substitutions on the B ring, enabling interaction with mutagens or modulation of metabolic enzymes involved in mutagen activation and detoxification.

Other extract constituents such as sesquiterpenes, even when present at low concentrations, also do not increase revertant colonies in the reverse mutation assay and may exhibit antimutagenic effects against DNA-damaging agents [52]. Similarly, coumarins, present at low levels in the extract, share this favorable safety profile and may also have contributed to the absence of genotoxic effects observed [53].

## 4. Material and Methods

### 4.1. Plant Material and Aqueous Extract Preparation

The trunk bark of *C. leptophloeos* was collected in January 2019 in the municipality of Buíque (Pernambuco, Brazil) at 8°34′14.9″ latitude and 37°14′37.3″ longitude. The specimen was deposited in the Herbarium of the Agronomic Institute of Pernambuco (IPA), Recife, Brazil, under voucher number IPA 84.037 and registered in SISGEN No. AA3B70A.

The collected bark samples were dried at room temperature (25 °C) for three weeks. Subsequently, a decoction was prepared to extract secondary metabolites by adding 7.5 g of fragmented barks to 150 mL of distilled water (1:20 *w*/*v*) [54] and boiling for 20 min. The resulting extract was lyophilized, yielding 5.76% dry weight, and stored at −20 °C.

### 4.2. Phytochemical Analysis

#### 4.2.1. Analysis by UPLC-MS/MS

For chemical evaluation, a 2 μL aliquot of the extract, previously solubilized in DMSO (Sigma-Aldrich, Saint Louis, MO, USA, 99.9%) at a concentration of 10 mg/mL, was analyzed using an ultra-performance efficiency liquid chromatography system coupled with tandem mass spectrometry (UPLC-MS/MS) in triplicate. The analysis was carried out on an Acquity H-Class UPLC chromatograph (Waters, Milford, MA, USA) coupled to a Bruker Impact II UHR-ESI-QqTOF mass spectrometer (Bruker Daltonics, Billerica, MA, USA).

For the chromatographic analysis, a BEH C18 reversed-phase column (1.7 μM, 2.1 mm × 100 mm, Waters, Milford, MA, USA), equipped with a compatible pre-column, was used. The mobile phase consisted of water (A), acetonitrile (B), and 2% formic acid (C). The gradient applied in the analytical method was as follows: 0–7.5 min, 5–45% B; 7.5–10 min, 45–95% B; 10–12 min, 95–100% B; followed by 12–15 min for column equilibration in the initial phase. Solvent C was maintained at a constant 5% (0.1% final concentration, except for 10–12 min), with a flow rate of 0.5 mL/min and a column temperature of 40 °C.

The mass spectrometer was operated in positive ion mode over a mass range of 30 to 1800 Da, with a data acquisition rate of 8 Hz. Instrument settings included an end plate offset of 500 Volts (V), a capillary voltage of 4500 V, nebulizer gas pressure of 4.0 bar, nitrogen drying gas flow at 10 L/min, and a drying temperature of 200 °C. MS/MS fragmentation was performed for the most intense ions, with a cycle time of 1 s and an absolute intensity threshold of 1500 counts per 1000 summed spectra. According to the MS^2^ fragmentation rules, mass ratios (*m*/*z*) below 200 Da were excluded. The “active exclusion” function was enabled to avoid repeated fragmentation of the same precursor ion. Precursor ions detected in more than three spectra were excluded from fragmentation, blocked for 0.3 min or until the “current intensity/previous intensity” was greater than or equal to 1.8.

Each run was automatically calibrated using 10 mM sodium formate (HCOONa) and the calibrated spectra were subsequently converted into mzXML files using DataAnalysis 4.0 and CompassXport **1.3** software.

#### 4.2.2. Dereplication of Natural Products of *Commiphora leptophloeos* Using NP^3^ MS Workflow Software

The annotation of secondary metabolites present in the aqueous extract of *C. leptophloeos* bark was performed using the automated software NP^3^ MS Workflow [55,56,57].

Data analysis was carried out using the software’s standard parameters for the pre-processing step, which involved the extraction of MS^1^ and MS^2^ data from *.mzXML files. During the main execution phase (run), clustering steps were applied to generate consensus spectra, based on separation by *m*/*z* values and retention times, along with quantification of peak areas derived from MS^1^ signal intensities in the samples. All standard parameters of the NP^3^ MS Workflow software were used, except for the retention time tolerance, which was adjusted to -t 10.10.

The process generated several output files, including: (a) an *.mgf file containing all consensus spectra, characterized primarily by MS^1^ (*m*/*z*), MS^2^ (fragmentation patterns), and mean retention time (RT); (b) a quantification table (*.csv) with the list of *m*/*z* (consensus spectra) in rows and columns with qualitative and quantitative properties, including peak areas, annotations, identifications, etc., referring to all *m*/*z* found in the sample set (in the generated *.mgf file); and (c) files used in the assembly of the molecular network, compatible with Cytoscape v. 3.9.1. By these files (list *.mgf), it was possible to annotate compounds using the GNPS platform (https://gnps.ucsd.edu/ProteoSAFe/status.jsp?task=2248366b7edf4a9fb84319243191a896, accessed on 15 June 2022) and add them to the quantification table *.csv (using the NP^3^ MS Workflow) for the final interpretation of the results, which now contain the chemical annotations from the UNPD-ISDB [58] and GNPS [59] databases. Additionally, molecular networking was performed using Cytoscape v. 3.9.1 based on the NP^3^-generated files.

### 4.3. In Vitro Antioxidant Activity

#### 4.3.1. DPPH Test

The DPPH assay was carried out according to [60]. The *C. leptophloeos* extract was dissolved in ethanol P.A. to prepare final concentrations of 0.78, 1.56, 3.12, 6.25, 12.5, 25, and 50 μg/mL. Ascorbic acid was used as the reference standard at the same concentrations. In a 96-well microplate, 50 μL of sample or standard solution was added to 150 μL of a 0.2 mM DPPH solution in each well. After 30 min of incubation in the dark, absorbance was measured at 515 nm using a microplate reader. Antioxidant activity was expressed as the percentage of DPPH reduction, calculated using the following formula:

I% = [(Ac − Aa)/Ac] × 100,(1)

where Ac is the absorbance of the control and Aa is the absorbance of the sample. Additionally, the EC_50_ value was estimated as described in [61].

#### 4.3.2. ABTS Test

The ABTS assay was performed according to [62]. To prepare the ABTS solution, 5 mL of ABTS (7 mM) was added to 88 μL of potassium persulfate (140 mM), both dissolved in dH_2_O. Subsequently, the resulting solution was adjusted to an absorbance of 0.700 ± 0.100 (at 734 nm) by diluting in ethanol P.A.

The *C. leptophloeos* extract was dissolved in ethanol P.A. to achieve final concentrations of 0.78, 1.56, 3.12, 6.25, 12.5, 25, and 50 μg/mL. Ascorbic acid was used as a standard at the same concentrations. In a 96-well microplate, 20 μL of sample or ascorbic acid and 220 μL of ABTS were added to each well. After 6 min of incubation, absorbance was measured at 734 nm using a microplate reader. Antioxidant activity was expressed as the percentage of ABTS reduction, calculated using the same formula as the DPPH test. Additionally, the EC_50_ value was estimated as described in [61].

#### 4.3.3. Phosphomolybdenum

The phosphomolybdenum assay, based on the reduction of molybdenum (VI) to molybdenum (V), was performed according to [63]. The *C. leptophloeos* extract was dissolved in distilled water to obtain final concentrations of 0.78, 1.56, 3.12, 6.25, 12.5, 25, 50, and 100 μg/mL. Ascorbic acid was used as a standard at the same concentrations. For the reaction, 1000 μL of phosphomolybdenum was added to 100 μL of the sample. The samples were then incubated in a thermostatic bath at 95 °C for 90 min. Absorbance was measured at 700 nm using a spectrophotometer.

Each reaction was conducted in triplicate, with a negative control (reagents and water). Antioxidant capacity was measured by the increase in absorbance, which indicates greater reduction power. The EC_50_ value, corresponding to an absorbance of 0.5 nm, was calculated by plotting the normalized absorbances at 695 nm against the corresponding concentrations [64].

#### 4.3.4. Reducing Power

The reductive power test was performed according to [65]. The *C. leptophloeos* extract was dissolved in water to obtain the final concentrations of 0.78, 1.56, 3.12, 6.25, 12.5, 25, 50, and 100 μg/mL. Ascorbic acid was used as a standard at the same concentrations. The reaction mixture consisted of 312.5 μL of phosphate buffer (0.2 M PBS; pH 6.6), 625 μL of potassium ferricyanide (1%), and 250 μL of the sample. The samples were incubated in a thermostatic bath at 50 °C for 30 min. Subsequently, 312.5 μL of trichloroacetic acid (10%) was added, and the samples were centrifuged at 1500 rpm for 10 min. Then, 312.5 μL of the supernatant was transferred to a new tube, followed by the addition of 312.5 μL of distilled water and 62.5 μL of ferric chloride (0.1%). Absorbance was measured at 700 nm using a spectrophotometer.

Reactions were conducted in triplicate, using distilled water as a negative control. Antioxidant capacity was measured by the increase in absorbance, indicating greater reducing power. The EC_50_ value, defined as the concentration at which the absorbance reaches 0.5 nm, was determined by plotting the normalized absorbance values at 700 nm against the corresponding concentrations [64].

### 4.4. Cytotoxicity, Genotoxicity, and Mutagenicity Tests

#### 4.4.1. Cell Lineage

For cytotoxicity and genotoxicity assays, the murine fibroblast cell line L929 was used. Cells were cultured in DMEM (Gibco^TM^, Grand Island, NY, USA), supplemented with fetal bovine serum (Gibco) and antibiotic–antimycotic solution (penicillin 10,000 Units/mL; streptomycin 10 μg/mL; and 25 μg/mL of amphotericin B). Culture flasks were maintained in an incubator with a humid atmosphere at 37 °C and 5% CO_2_.

#### 4.4.2. MTT Test (Cytotoxicity)

The MTT test was performed according to [66], using 96-well plates seeded with 2 × 10^4^ cells per well. Cells were exposed for 24 h at 37 °C in 5% CO_2_ at 13 concentrations (1.56, 3.12, 6.25, 12.5, 25, 50, 100, 200, 400, 800, 1600, 3200, and 6400 μg/mL) of the *C. leptophloeos* bark extract, as recommended by [67]. After exposure, cells were incubated with MTT tetrazolium salt for 3 h under the same conditions. Formazan crystals were solubilized in DMSO, and absorbance was measured at 540 nm using a spectrophotometer. The experiment was carried out with five replicates.

#### 4.4.3. Micronucleus Test with Cytokinesis Block (CBMN Test, Genotoxicity)

The in vitro CBMN test was performed to evaluate the safety of the *C. leptophloeos* bark extract, following the protocol described by [19], with minor modifications. After 24 h of stabilization, 2 × 10^6^ cells per 75 cm^2^ flask were exposed to the extract at concentrations of 6.25, 25, 100, and 400 μg/mL. Methyl methanesulfonate (MMS; 4 × 10^−4^ M) was used as the positive control (PC), and culture medium alone served as the negative control (NC). The tested concentrations were selected based on the MTT assay results: 400 μg/mL, which showed significantly higher viability than 100%, and 6.25, 25, and 100 μg/mL, which showed statistically equivalent viability to 100%. After 48 h of exposure, treatment solutions were replaced with fresh culture medium containing 3 μg/mL cytochalasin. After an additional 28 h of incubation, cells were fixed in Carnoy (methanol:acetic acid; 9:1), transferred to slides, and stained with DAPI.

The experiment was performed in triplicate (*n* = 3). For each flask, 3000 binucleated cells were analyzed (1000 cells per slide), totaling 9000 cells per treatment. Each replicate represents the average of three slides per flask. The frequencies of micronuclei (MN), nuclear buds (NBs), and nucleoplasmic bridges (NPBs) were quantified. The Cytokinesis-Block Proliferation Index (CBPI) was calculated according to the [67] guideline following the formula CBPI = [M1 + 2M2 + 3 (M3 + M4)]/N, where N is the total number of cells analyzed and M1 to M4 represent the number of cells with one to four nuclei. For CBPI determination, 1500 cells were evaluated per replicate, totaling 4500 cells per treatment.

#### 4.4.4. Ames/Salmonella Test (Pre-Incubation Method, Mutagenicity)

The Ames/*Salmonella* test (reverse mutation test) was performed using the pre-incubation method with *S. typhimurium* strains TA98 and TA100 following the protocol described by [68]. A preliminary cytotoxic assessment was carried out at concentrations of 6.25, 25, 100, 400, 800, 1000, 1200, and 1600 μg/plate to evaluate the toxicity of the compounds on strain TA100, both in the presence and absence of metabolic activation (Aroclor 1254). Distilled water was used as a negative control. Sodium azide and 4-nitroquinoline-1-oxide were used as positive controls for strains TA100 and TA98, respectively, while 2-aminoanthracene was used as the positive control for both strains under metabolic activation. For the exposure step, 100 μL of the sample was added to 100 μL of bacterial culture and 500 μL of S9 mix or phosphate buffer in test tubes. Then, the tubes were pre-incubated at 37 °C with shaking (170 rpm) for 30 min. After pre-incubation, 2.5 mL of top agar was added to each tube and poured into Petri dishes with minimal agar medium. Plates were incubated at 37 °C for 72 h.

Preliminary results indicated that the evaluated concentrations did not induce cytotoxicity. Therefore, the definitive test was conducted using *S. typhimurium* strains TA100 and TA98 at concentrations of 6.25, 25, 100, 400, and 1600 μg/plate, both in the presence and absence of metabolic activation (S9 mix induced with Aroclor 1254). After exposure, the number of revertant colonies was counted. The Mutagenicity Index (MI), defined as the ratio of the number of revertants in the treated group to the number in the negative control, was calculated for each concentration using the following formula:

$$MI\;\left(\%\right)=\frac{number\;of\;revertants\;\left(compound\;test\right)}{number\;of\;revertants\;\left(negative\;control\right)}$$

Tested concentrations that showed MI ≥ 2, along with a statistically significant increase in the number of revertant colonies compared to the negative control, were considered indicative of mutagenic potential [69].

### 4.5. Statistical Analysis

Results were expressed as mean ± standard deviation. The antioxidant assays (DPPH, ABTS, phosphomolybdenum, and reducing power), as well as the micronucleus and reverse mutation assays, were performed in triplicate, while the MTT assay was conducted in quintuplicate. Data on antioxidant potential were assessed for normality using the Shapiro–Wilk test and for homogeneity of variance using Levene’s test, both of which confirmed the assumptions for parametric analysis. These data were then compared using the parametric Student’s *t*-test. In contrast, data from cytotoxicity, micronucleus, and CBPI assays did not meet assumptions of normality or homogeneity. Therefore, they were analyzed using the non-parametric Kruskal–Wallis test. All statistical analyses were performed using Statistica 13.3 software. Differences were considered statistically significant at *p* < 0.05. EC_50_ values were estimated by fitting a non-linear regression curve using the formula *Y* = 100/(1 + 10^(*LogEC_50_* − X)), with concentrations on a base-10 logarithmic scale based on normalized responses [70,71]. This analysis was performed using GraphPad Prism 5.0 software.

## 5. Conclusions

*Commiphora leptophloeos* bark is traditionally used for respiratory conditions such as cough and bronchitis, possibly associated with oxidative stress in the airways. Although this study did not include direct anti-inflammatory assays, the strong in vitro antioxidant activity suggests a potential mechanism for reducing oxidative damage in airways. The results demonstrated antioxidant activity comparable to ascorbic acid in the DPPH assay. Furthermore, the active concentrations showed no cytotoxicity or genotoxicity in L929 murine fibroblasts and no genotoxic effects in *S. typhimurium* strains TA98 and TA100. Phytochemical analysis revealed a predominance of organooxygen compounds and flavonoids, with procyanidin B2 as the major constituent. These findings support the traditional medicinal use of *C. leptophloeos* bark, highlighting its chemical profile and safety at the tested concentrations, and reinforce the importance of this species in ethnobotanical conservation. Further in vivo and pre-clinical studies are recommended to validate its therapeutic potential.

## Figures and Tables

**Table 1:** List of annotated spectra in the SSMN [M+H]^+^ of the aqueous extract of *Commiphora leptophloeos*. Compound annotation was performed using the GNPS and UNPD-ISDB databases.

No	*m*/*z*	Rt (s)	Annotated Compound	Class GNPS	Formula	MolecularMass	CAS
**8**	303.1077	94,44	Picraquassioside D	Organooxygen	C_13_H_18_O_8_	302.1002	148707-37-3
**11**	579.1450	123,07	Procyanidin B2	Flavonoid	C_30_H_26_O_12_	578.52	82262-99-5
**12**	466.1927	137,46	(3,4-dimethoxyphenoxy)-6-[(3,4,5-trihydroxyoxan-2-yl)oxymethyl]oxane-3,4,5-triol	Organooxygen	C_19_H_28_O_12_	448.1581	-
**14**	579.1505	146,20	ent-epicatechin-(4α→8)-ent-epicatechin	Flavonoid	C_30_H_26_O_12_	578.1424	82262-99-5
**15**	355.1027	149,38	Magnolioside	Cumarin	C_16_H_18_O_9_	354.0950	20186-29-2
**18**	347.1340	158,40	1-beta-D-glucopyranosyloxy-3,4,5-trimethoxybenzene	Organooxygen	C_15_H_22_O_9_	346.1264	41514-64-1
**19**	579.1450	161,75	Procyanidin B2	Flavonoid	C_30_H_26_O_12_	578.52	82262-99-5
**21**	477.1976	165,17	Forsythoside E	Organooxygen	C_21_H_32_O_12_	476.1894	31864-08-09
**22**	493.1923	169,41	1-(alpha-L-rhamnopyranosyl-(1->6)-beta-D-glucopyranosyloxy)-3,4,5-trimethoxybenzene	Flavonoid	C_21_H_32_O_13_	492.1843	1023336-03-9
**23**	387.202	187,37	(3S,5R,8R)-3,5-dihydroxy-6,7-megastigmadien-9-one 5-O-beta-D-glucopyranoside	Fatty acyl	C_19_H_30_O_8_	386.437	120278-09-3
**28**	579.1450	236,07	Procyanidin B2	Flavonoid	C_30_H_26_O_12_	578.52	82262-99-5
**29**	625.1774	261,04	isorhamnetin 3-O-neohesperidoside	Unclassified	C_28_H_32_O_16_	624.1690	55033-90-4
**30**	219.1018	262,89	(+)-(E,E)-3-Hydroxy-7-phenyl-4,6-heptadienic acid	Diarylpropanoid	C_13_H_14_O_3_	218.0943	-
**32**	453.1407	269,81	6′-O-vanilloyltachioside	Organooxygen	C_21_H_24_O_11_	452.409	1413911-15-9
**33**	359.1492	285,96	Glicophenone	Lignan	C_20_H_22_O_6_	358.385	111254-27-4
**34**	359.1495	301,13	Glicophenone	Lignan	C_20_H_22_O_6_	358.385	111254-27-4
**38**	295.1028	339,42	3-O-beta-D-glucopyranosyl-2-deoxy-D-ribono-gamma-lactone	Organooxygen	C_11_H_18_O_9_	294.0951	959761-28-5
**41**	221.1904	390,55	Helianthol A	Organonitrogen	C_15_H_24_O	220.1827	72916-06-4
**43**	331.0816	426,04	Butoletol	Cumarin	C_17_H_14_O_7_	330.0740	35214-88-1
**45**	403.2025	499,66	Aurantiamide	Carboxylic acid	C_25_H_26_N_2_O_3_	402.1943	58115-31-4
**48**	445.2134	555,18	Benzenepropanamide, N-[2-(acetyloxy)-1-(phenylmethyl)ethyl]-α-(benzoylamino)	Carboxylic acid	C_27_H_28_N_2_O_4_	444.2049	56121-42-7
**49**	229.1226	597,67	3,7-dimethyl-5-isopropyl-6-formylindenone	Unclassified	C_15_H_16_O_2_	228.1150	-

No: compound number. *m*/*z*: mass charge ratio of the ion shown as the [M+H]^+^ species. Rt: retention time.

**Table 2:** Effective concentration (EC_50_) of the aqueous extract bark of *Commiphora leptophloeos* in in vitro antioxidant assays.

Sample	EC_50_ (μg/mL) ^1^			
	DPPH ^2^	ABTS ^2^	Phosphomolybdenum ^3^	Reducing Power ^3^
*C. leptophloeos*	5.43 ± 1.12 a	12.40 ± 1.15 a	35.20 ± 1.16 a	31.27 ± 1.25 a
Ascorbic acid	6.40 ± 1.32 a	5.48 ± 1.25 b	12.70 ± 1.18 b	6.39 ± 1.10 b

^1^ EC_50_ values expressed in μg/mL of antioxidant activity. Different letters in the columns indicate statistical differences by the *t*-test (*p* < 0.05). ^2^ Effective concentrations required to reduce 50% of free radicals. ^3^ Effective concentration at which the absorbance reaches 0.5 nm. The EC_50_ were calculated using the non-linear regression model Y = 100/(1 + 10^(LogEC50 − X)^) in GraphPad Prism 5.0.

**Table 3:** Nuclear alterations in murine fibroblasts (L929 cell line) after 48 h of exposure to the aqueous extract of *Commiphora leptophloeos* bark.

Treatment μg/mL	MN *	BN *	PN *	Total Alterations *	CBPI
NC	10.00 ± 2.68 a	8.00 ± 2.60 a	0.00 ± 0.00 a	18.00 ± 4.33 a	1.70 ab
PC	66.00 ± 7.78 b	25.60 ± 5.55 b	1.20 ± 0.40 a	92.43 ± 8.92 b	1.42 c
6.25	8.22 ± 4.20 a	7.88 ± 2.80 a	1.20 ± 0.40 a	16.55 ± 4.27 a	1.72 a
25	9.11 ± 4.64 a	10.00 ± 3.35 a	1.40 ± 0.80 a	20.51 ± 8.34 ac	1.69 ab
100	8.25 ± 2.31 a	9.22 ± 2.58 a	1.00 ± 0.00 a	18.00 ± 5.37 a	1.66 ab
400	15.65 ± 4.21 c	8.16 ± 2.11 a	1.00 ± 0.00 a	21.55 ± 7.87 ac	1.69 ab

NC: Negative Control (culture medium only); PC: Positive Control (methyl methanesulfonate; 4 × 10^−4^ M). MN: micronucleus; BN: nuclear shoots; PN: nucleoplasmic bridge. CBPI: Cytokinesis-Block Proliferation Index. * Average value observed per 1000 cells. Means with different letters in the same column indicate statistically differences from the negative control by the Kruskal–Wallis test (*p* < 0.05).

**Table 4:** Mean number of revertant colonies per plate of *Salmonella typhimurium* strains TA100 and TA98, with and without S9 metabolic activation, after exposure to aqueous extract of *Commiphora leptopholeos* bark.

Treatment	Concentration (μg/plate)	−S9	MI	+S9	MI
TA100					
NC	0	100.60 ± 9.0 a	-	115.40 ± 11.6 a	-
NaN_3_	0.5	1023.50 ± 55.9 b	7.9	-	-
2AA	5	-	-	330.00 ± 100.0 b	3.7
*C. leptopholeos*	6.25	102.30 ± 8.5 a	0.8	98.70 ± 7.0 a	1.1
	25	82.00 ± 4.6 a	0.6	117.70 ± 10.7 a	1.3
	100	104.30 ± 14.6 a	0.8	102.30 ± 9.3 a	1.1
	400	111.00 ± 13.1 a	0.9	99.30 ± 8.3 a	1.1
	1600	98.00 ± 6.2 a	0.8	100.00 ± 18.2 a	1.1
TA98					
NC	0	24.20 ± 5.12 a	-	31.25 ± 1.71 a	-
4NQO	0.5	109.50 ± 16.26 b	1.9	-	-
2AA	5	-	-	281.00 ± 18.38 b	8.4
*C. leptopholeos*	6.25	21.67 ± 2.08 a	0.4	32.33 ± 4.73 a	0.6
	25	21.00 ± 2.83 a	0.4	31.00 ± 4.58 a	0.8
	100	26.00 ± 2.65 a	0.5	34.67 ± 8.39 a	1.0
	400	18.00 ± 2.83 a	0.3	29.33 ± 2.08 a	0.8
	1600	24.00 ± 6.93 a	0.4	31.33 ± 4.93 a	0.9

NC: Negative Control (distilled water only); NaN_3_: Sodium azide. 4NQO: 4-nitroquinoline-1-oxide. 2AA: 2-aminoanthracene. Different letters in the same column have mean values and standard deviations that are statistically different from the negative control by the Mann–Whitney test (*p* < 0.05). MI, Mutagenicity Index: number of revertant colonies per treated plate divided by the number of revertant colonies per negative control plate.

## Data Availability

Data are contained within the article and the Supplementary Materials. Further inquiries can be directed to the corresponding author.

## Supplementary Materials

The following supporting information can be downloaded at: https://www.mdpi.com/article/10.3390/ph18060863/s1. Supplementary Figure S1. Spectra Similarity Molecular Networking (SSMN) generated from the aqueous extract of *Commiphora leptophloeos* using UPLC-MS/MS. A) Raw SSMN (includes all distinct ions processed through the NP3 MS Workflow); B) [M+H]^+^ SSMN (includes selected ions identified as protonated compounds). Each consensus spectrum (MS/MS fragmentation pattern) is represented as a node (circle or square) in the SSMN. The values below the geometric shapes (omitted in panel A) indicate the mass-to-charge ratio (*m/z*—mzConsensus). Edges connecting the nodes within components (also referred to as “clusters”) represent the similarity between pairs of consensus spectra. Thicker edges indicate higher similarity between the fragmentation patterns. Node color corresponds to the *m/z* value, ranging from 200 Da (yellow) to 867 Da (dark blue). Black circular borders denote spectra with no matches in the consulted databases. Fuchsia circular borders indicate annotations from the “Universal Natural Products in Silico Database” (UNPD-ISDB). Black square borders represent identifications via the “Global Natural Products Social Molecular Networking” (GNPS), while fuchsia square borders indicate annotations from both UNPD-ISDB and GNPS. Dashed fuchsia borders denote weak chemical annotations from the UNPD database (MQScore < 0.4). The font color of the *m/z* values also reflects annotation quality: blue indicates a GNPS mzErrorppm > 20, while bold black denotes a strong annotation with GNPS MQScore > 0.9. Supplementary Figure S2. Chromatographic profile obtained by UPLC-MS/MS analysis of the aqueous extract of Commiphora *leptophloeos*. A) Chromatograms acquired for each replicate; B) Enlarged chromatogram highlighting the retention time (RT) region from 1 to 6 min. Peaks labeled with RT values correspond to the major constituents of the sample. The black trace represents the UV chromatogram (190–600 nm), while the green trace corresponds to the base peak chromatogram (200–1800 Da). Supplementary Figure S3. Proposed fragmentation pathway for ions annotated as Procyanidin B2. (A) MS/MS fragmentation spectra obtained for the ion at *m/z* 579.1450 at two distinct retention times: RT = 161.75 s and RT = 236.07 s. (B) Proposed fragmentation scheme for the compound annotated as Procyanidin B2, correlating the major fragment ions observed in the spectra. The MS/MS spectra exhibit a high degree of qualitative similarity, differing primarily in the relative intensities of certain fragment ions. This observation suggests that both signals likely originate from the same core molecular scaffold. However, the chromatographic separation of the peaks implies the presence of stereochemical differences between the two species, which are sufficient to alter their retention behavior under the chromatographic conditions employed. Supplementary Table S1. Classification of main metabolites of the aqueous extract of *Commiphora leptophloeos* annotated with the UNPD-ISDB database. Consensus spectra used were from the SSMN [M+H]^+^. Rt: retention time. Supplementary Table S2. Annotation parameters obtained from GNPS database for selected ions in *Commiphora leptophloeos* extract. No: compound number. m/z: mass charge ration of the ion pointed as the [M+H]^+^ species. Rt: retention time. 1Annotation confidence: classification based in parameters mzPPMError, MQScore and NumSharedPeaks. “High”—mzPPMError < 20; MQScore > 0.9 and NumSharedPeaks > 6; “Average”—mzPPMError < 20; 0.8 < MQScore < 0.9 and NumSharedPeaks > = 6; “Low”—mzPPMError > 20.

## Abbreviations

The following abbreviations are used in this manuscript:

ABTS	2,2′-Azino-bis(3-ethylbenzothiazoline-6-sulfonic acid)
CBMN	Micronucleus test with cytokinesis block
CBPI	Cytokinesis-Block Proliferation Index
CNPq	Conselho Nacional de Desenvolvimento Científico e Tecnológico
DPPH	2,2-Diphenyl-1-picrylhydrazyl
EC_50_	Effective concentration
EMA	European Medicines Agency
FDA	Food and Drug Administration
GNPS	Global Natural Products Social Molecular Networking
MI	Mutagenicity Index
MN	Micronuclei
MTT	(3-(4,5-Dimethylthiazol-2yl)-2,5-diphenyltetrazolium bromide)
NB	Nuclear bud
NC	Negative control
NPB	Nucleoplasmic bridge
PC	Positive control
SSMN	Spectra Similarity Molecular Networking
UPLC-MS/MS	Ultra-performance liquid chromatography coupled with tandem mass spectrometry
UNPD	Universal Natural Products in Silico Database
