# Supplementary material for: Commiphora leptophloeos Bark Decoction: Phytochemical Composition, Antioxidant Capacity, and Non-Genotoxic Safety Profile
Source: Pharmaceuticals (Basel). 2025 Jun 10;18(6):863. doi: 10.3390/ph18060863 (PMC12196306; doi:10.3390/ph18060863)
Supplement: Supplementary file 1 [file pharmaceuticals-18-00863-s001.zip › Supplementary Table S2.pdf]

Supplementary Table S2. Annotation parameters obtained from GNPS database for selected ions in *Commiphora leptophloeos* extract.

No.	*m/z*	Rt (sec)	Annotated Compound	Peak area	gnps_SpectrumID	mzErrorPPM (gnps)	MQScore (gnps)	NumSharedPeaks (gnps)	Annotatoion Confidence[1]
8	303.1077	94.44	(3-hydroxy-5-methoxy-phenyl)-beta-D-glucopyranoside	4190.00	CCMSLIB00000853124	2.42	0.976	7.0	High
11	579.1505	123.07	ent-epicatechin-(4alpha->8)- ent-epicatechin	4556.00	CCMSLIB00003138102	9.38	0.938	14.0	High
12	466.1927	137.46	2-(3,4-dimethoxyphenoxy)-6-[(3,4,5-trihydroxyoxan-2-yl)oxymethyl]oxane-3,4,5-triol	3571.67	CCMSLIB00000847914	1.57	0.834	12.0	Average
14	579.1505	146.20	Procyanidin B2	5502.00	CCMSLIB00003139020	0.74	0.887	16.0	Average
15	355.1027	149.38	5,7-dihydroxy-3-methylchromone-7-O-beta-D-glucoside	3631.67	CCMSLIB00000848608	271052	0.957	5.0	Low
18	347.134	158.40	1-beta-D-glucopyranosyloxy-3,4,5-trimethoxybenzene	2730.33	CCMSLIB00000845174	2.99	0.873	6.0	Average
19	579.1499	161.75	Procyanidin B2	135402.00	CCMSLIB00003138102	8.43	0.937	14.0	High
21	477.1976	165.17	Forsythoside E	2594.33	CCMSLIB00004698066	30261.7	0.814	11.0	Low
22	493.1923	169.41	1-(alpha-L-rhamnopyranosyl-(1->6)-beta-D-glucopyranosyloxy)-3,4,5-trimethoxybenzene	4425.00	-	193024	0.794669	13.0	Low
23	387.202	187.37	(3S,5R,8R)-3,5-dihydroxy-6,7-megastigmadien-9-one 5-O-beta-D-glucopyranoside	1580.00	CCMSLIB00000851547	2.52	0.877464	8.0	Average

28	579.1503	236.07	Procyanidin B2	9941.33	CCMSLIB00003137035	0.421	0.902629	16.0	High
29	625.1774	261.04	isorhamnetin 3-O-neohesperidoside	3981.33	CCMSLIB00003139822	15.32	0.978887	13.0	High
30	219.1018	262.89	(+)-(E,E)-3-Hydroxy-7-phenyl-4,6-heptadienic acid	3337.00	-	113199	0.769	7.0	Low
32	453.1407	269.81	6'-O-vanilloyltachioside	1479.33	-	113001	0.766	7.0	Low
33	359.1492	285.96	glicophenone	3293.00	CCMSLIB00000851650	0.68	0.965	14.0	High
34	359.1495	301.13	glicophenone	1866.00	CCMSLIB00000851650	1.44	0.952	8.0	High
38	295.1028	339,42	3-O-β-D-glucopyranosyl-2-deoxy-D-ribono-gamma-lactone	2529.00	CCMSLIB00004712562	384159.00	0.916	9.0	Low
41	221.1904	390,55	Helianthol A	5438.33	CCMSLIB00000567926	227642.00	0.756	5.0	Low
43	331.0816	426,04	3,5,7-trihydroxy-6,4'-dimethoxyflavone	2166.00	CCMSLIB00005740591	497795.00	0.859	5.0	Low
45	403.2025	499,66	Aurantiamide	2125.33	CCMSLIB00000854777	94358.50	0.860	10.0	Low
48	445.2134	555,18	Benzenepropanamide, N-[2-(acetyloxy)-1-(phenylmethyl)ethyl]-α-(benzoylamino)	7078.67	CCMSLIB00000854777	3.15	0.914	13.0	High
49	229.1226	597,67	3,7-dimethyl-5-isopropyl-6-formylindenone	4329.33	CCMSLIB00000085376	139148.00	0.761	5.0	Low

No: compound number. *m/z*: mass charge ration of the ion pointed as the $[M+H]^+$ species. Rt: retention time. [1]Annotation confidence: classification based in parameters mzPPMError, MQScore and NumSharedPeaks. “High” - mzPPMError < 20; MQScore > 0,9 and NumSharedPeaks > 6; “Average” - mzPPMError < 20; 0,8 < MQScore < 0,9 and NumSharedPeaks > = 6; “Low” - mzPPMError > 20.
