# Supplementary material for: Commiphora leptophloeos Bark Decoction: Phytochemical Composition, Antioxidant Capacity, and Non-Genotoxic Safety Profile
Source: Pharmaceuticals (Basel). 2025 Jun 10;18(6):863. doi: 10.3390/ph18060863 (PMC12196306; doi:10.3390/ph18060863)
Supplement: Supplementary file 1 [file pharmaceuticals-18-00863-s001.zip › Supplementary Figure S1.pdf]

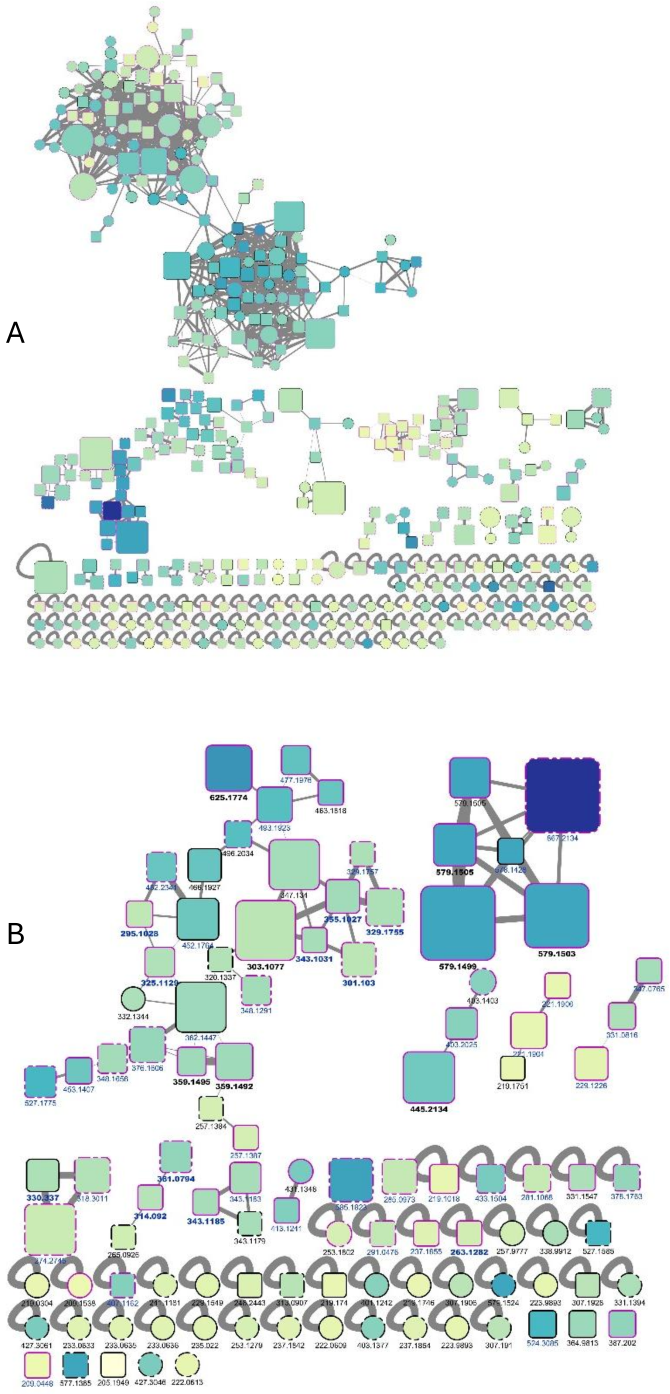

Supplementary Figure S1. Spectra Similarity Molecular Networking (SSMN) generated from the aqueous extract of *Commiphora leptophloeos* using UPLC-MS/MS. A) Raw SSMN (includes all distinct ions processed through the NP[3] MS Workflow); B) $[M+H]^+$ SSMN (includes selected ions identified as protonated compounds).

Each consensus spectrum (MS/MS fragmentation pattern) is represented as a node (circle or square) in the SSMN. The values below the geometric shapes (omitted in panel A) indicate the mass-to-charge ratio (*m/z* - mzConsensus). Edges connecting the nodes within components (also referred to as “clusters”) represent the similarity between pairs of consensus spectra. Thicker edges indicate higher similarity between the fragmentation patterns. Node color corresponds to the *m/z* value, ranging from 200 Da (yellow) to 867 Da (dark blue). Black circular borders denote spectra with no matches in the consulted databases. Fuchsia circular borders indicate annotations from the “Universal Natural Products in Silico Database” (UNPD-ISDB). Black square

borders represent identifications via the “Global Natural Products Social Molecular Networking” (GNPS), while fuchsia square borders indicate annotations from both UNPD-ISDB and GNPS. Dashed fuchsia borders denote weak chemical annotations from the UNPD database (MQScore < 0.4). The font color of the *m/z* values also reflects annotation quality: blue indicates a GNPS mzErrorppm > 20, while bold black denotes a strong annotation with GNPS MQScore > 0.9.
