# Supplementary material for: Commiphora leptophloeos Bark Decoction: Phytochemical Composition, Antioxidant Capacity, and Non-Genotoxic Safety Profile
Source: Pharmaceuticals (Basel). 2025 Jun 10;18(6):863. doi: 10.3390/ph18060863 (PMC12196306; doi:10.3390/ph18060863)
Supplement: Supplementary file 1 [file pharmaceuticals-18-00863-s001.zip › Supplementary Figure S2.pdf]

A

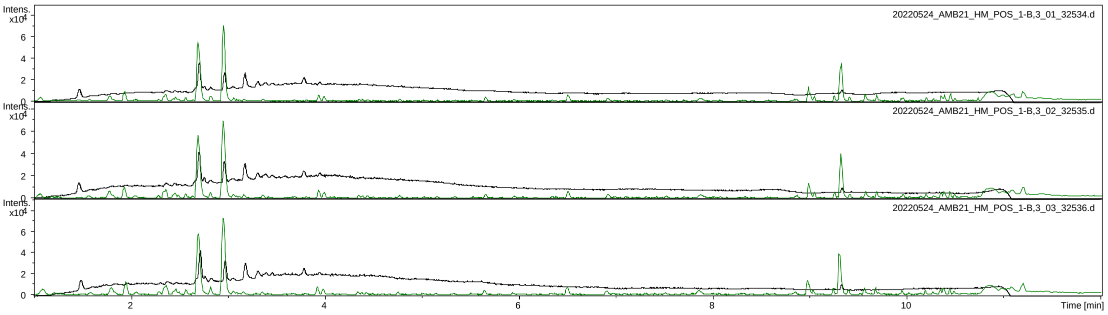

B

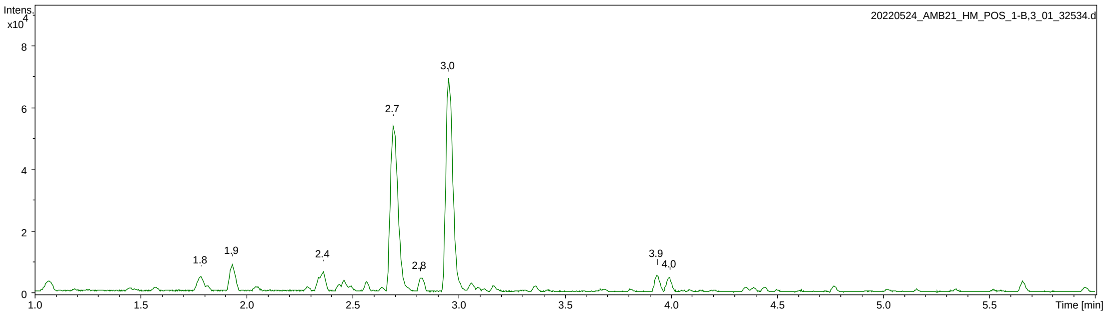

Supplementary Figure S2. Chromatographic profile obtained by UPLC-MS/MS analysis of the aqueous extract of *Commiphora leptophloeos*. A) Chromatograms acquired for each replicate; B) Enlarged chromatogram highlighting the retention time (RT) region from 1 to 6 minutes. Peaks labeled with RT values correspond to the major constituents of the sample. The black trace represents the UV chromatogram (190–600 nm), while the green trace corresponds to the base peak chromatogram (200–1800 Da).
