# Supplementary material for: Commiphora leptophloeos Bark Decoction: Phytochemical Composition, Antioxidant Capacity, and Non-Genotoxic Safety Profile
Source: Pharmaceuticals (Basel). 2025 Jun 10;18(6):863. doi: 10.3390/ph18060863 (PMC12196306; doi:10.3390/ph18060863)
Supplement: Supplementary file 1 [file pharmaceuticals-18-00863-s001.zip › Supplementary Figure S3.pdf]

A

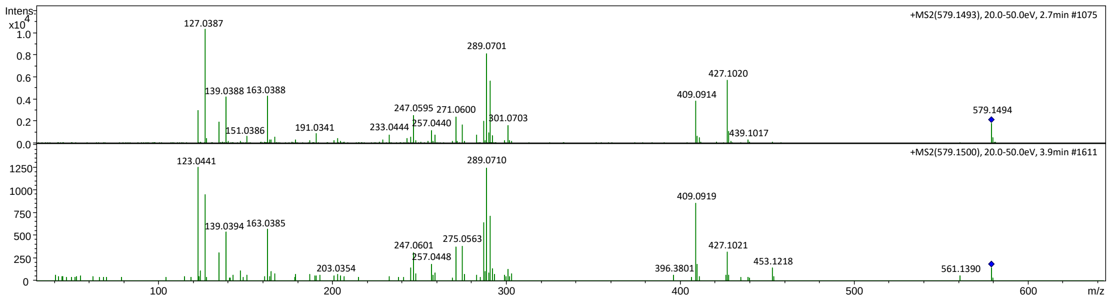

B

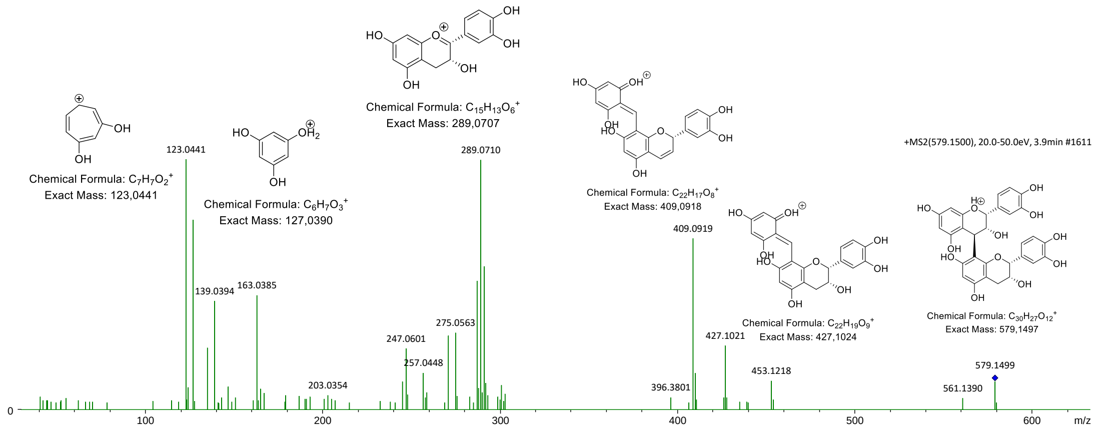

Supplementary Figure S3. Proposed fragmentation pathway for ions annotated as Procyanidin B2. A) MS/MS fragmentation spectra obtained for the ion at *m/z* 579.1450 at two distinct retention times: RT = 161.75 seconds and RT = 236.07 seconds. B) Proposed fragmentation scheme for the compound annotated as Procyanidin B2, correlating the major fragment ions observed in the spectra. The MS/MS spectra exhibit a high degree of qualitative similarity, differing primarily in the relative intensities of certain fragment ions. This observation suggests that both signals likely originate from the same core molecular scaffold. However, the chromatographic separation of the peaks implies the presence of stereochemical differences between the two species, which are sufficient to alter their retention behavior under the chromatographic conditions employed.
