# Supplementary material for: Commiphora leptophloeos Bark Decoction: Phytochemical Composition, Antioxidant Capacity, and Non-Genotoxic Safety Profile
Source: Pharmaceuticals (Basel). 2025 Jun 10;18(6):863. doi: 10.3390/ph18060863 (PMC12196306; doi:10.3390/ph18060863)
Supplement: Supplementary file 1 [file pharmaceuticals-18-00863-s001.zip › Supplementary Table S1.pdf]

Supplementary Table S1. Classification of main metabolites of the the aqueous extract of *Commiphora leptophloeos* annotated with the UNPD-ISDB database. Consensus spectra used were from the SSMN $[M+H]^+$. Rt: retention time.

$[M+H]^+$	Rt (seconds)	Super Class	Class
381,0794	29,295	Lignans|Lignans, neolignans and related compounds	Arylnaphthalene and aryltetralin lignans|Arylnaphthalene lignans
325,1129	30,7575	Saccharides|Organic oxygen compounds	Disaccharides|Organooxygen compounds
291,0476	31,5601	Chromanes|Benzenoids	Chromones|Benzene and substituted derivatives
301,103	36,9278	Phenolic acids (C6-C1)|Organic oxygen compounds	Simple phenolic acids|Organooxygen compounds
348,1291	72,1031	Organic acids and derivatives	Carboxylic acids and derivatives
281,1068	75,5798	Chromanes|Organoheterocyclic compounds	Chromones|Benzopyrans
303,1077	94,4411	Phloroglucinols|Organic oxygen compounds	Acyl phloroglucinols|Organooxygen compounds
585,1823	109,1058	Flavonoids|Phenylpropanoids and polyketides	Dihydroflavonols|Flavonoids
579,1505	123,0678	Flavonoids|Phenylpropanoids and polyketides	Proanthocyanins|Flavonoids
348,1656	140,1702	Peptide alkaloids;Linear polyketides|Benzenoids;	Open-chain polyketides
579,1505	146,1965	Flavonoids|Phenylpropanoids and polyketides	Proanthocyanins|Flavonoids
355,1027	149,3816	Chromanes|Organic oxygen compounds	Chromones|Organooxygen compounds
378,1763	150,3536	Small peptides|Organic acids and derivatives	Aminoacids|Carboxylic acids and derivatives
376,1606	154,0503	Tyrosine alkaloids|Benzenoids	Phenylethylamines|Benzene and substituted derivatives
347,134	158,3989	Organic oxygen compounds	Organooxygen compounds
579,1499	161,7527	Flavonoids|Phenylpropanoids and polyketides	Proanthocyanins|Flavonoids
477,1976	165,1666	Phenylethanoids (C6-C2)|Organic oxygen compounds	Phenylethanoids|Organooxygen compounds
493,1923	169,4062	Organic oxygen compounds	Organooxygen compounds
387,202	187,3678	Apocarotenoids|Lipids and lipid-like molecules	Megastigmanes|Prenol lipids
867,2134	189,6402	Flavonoids|Phenylpropanoids and polyketides	Proanthocyanins|Flavonoids
285,0973	201,6	Lignans|Benzenoids	Neolignans|Phenols
527,1775	228,4916	Aromatic polyketides|Phenylpropanoids and polyketides	Depsides|Depsides and depsidones
579,1503	236,0705	Flavonoids|Phenylpropanoids and polyketides	Proanthocyanins|Flavonoids
625,1774	261,0379	Flavonoids|Phenylpropanoids and polyketides	Flavonols|Flavonoids
219,1018	262,8874	Phenylpropanoids (C6-C3)|Organic acids and derivatives	Cinnamic acids and derivatives|Hydroxy acids and derivatives
453,1407	269,8072	Phenolic acids (C6-C1)|Phenylpropanoids and polyketides	Simple phenolic acids|Tannins

$[M+H]^+$	Rt (seconds)	Super Class	Class
359,1492	285,9626	Flavonoids|Phenylpropanoids and polyketides	Chalcones|Stilbenes
359,1495	301,1346	Flavonoids|Phenylpropanoids and polyketides	Chalcones|Stilbenes
331,1547	320,4873	Monoterpenoids|Lipids and lipid-like molecules	Pinane monoterpenoids|Prenol lipids
343,1185	330,975	Phenylpropanoids (C6-C3)|Organic oxygen compounds	Cinnamic acids and derivatives|Organooxygen compounds
462,2341	339,2163	Organic oxygen compounds	Organooxygen compounds
295,1028	339,4201	Organic oxygen compounds	Organooxygen compounds
343,1183	357,0387	Phenylpropanoids (C6-C3)|Organic oxygen compounds	Cinnamic acids and derivatives|Organooxygen compounds
253,1802	380,4917	Sesquiterpenoids|Lipids and lipid-like molecules	Germacrane sesquiterpenoids|Prenol lipids
221,1904	390,5527	Sesquiterpenoids|Lipids and lipid-like molecules	Bisabolane sesquiterpenoids|Prenol lipids
433,1504	419,1476	Organic acids and derivatives	Carboxylic acids and derivatives
331,0816	426,0393	Flavonoids|Phenylpropanoids and polyketides	Flavonols|Flavonoids
403,1403	498,247	Flavonoids|Phenylpropanoids and polyketides	Dihydroflavonols|Flavonoids
403,2025	499,6573	Peptide alkaloids	Dipeptides
274,2745	539,0937	Lysine alkaloids|Organoheterocyclic compounds	Piperidine alkaloids|Quinolidines
318,3011	542,8542	Lysine alkaloids|Alkaloids and derivatives	Quinolizidine alkaloids|Lupin alkaloids
229,1226	597,6659	Sesquiterpenoids|Benzenoids	Cadinane sesquiterpenoids|Indenes and isoindenes
329,1755	616,6165	Flavonoids|Phenylpropanoids and polyketides	Flavans|Flavonoids
